# Immune and Inflammation Markers as a Predictor of Overall Survival in Patients with Hematologic Malignancies: A Retrospective Cohort Study

**DOI:** 10.3390/medicina61061019

**Published:** 2025-05-30

**Authors:** Mehmet Ali Ucar, Anıl Tombak, Aydın Akdeniz, Hüseyin Derya Dinçyürek, Meryem Şener, Mahmut Bakır Koyuncu, Eyüp Naci Tiftik, Recep Dokuyucu

**Affiliations:** 1 Department of Hematology, Faculty of Medicine, Balcalı Hospital, Cukurova University, Adana 01330, Turkey; mucardr@hotmail.com.tr (M.A.U.); hdincyurek@cu.edu.tr (H.D.D.); dr.meryemsener@gmail.com (M.Ş.); 2 Department of Internal Medicine, Division of Hematology, Faculty of Medicine, Mersin University, Mersin 33110, Turkey; aniltombak@hotmail.com (A.T.); akdenizdr@mersin.edu.tr (A.A.); drmbkoyuncu@gmail.com (M.B.K.); ntiftik@mersin.edu.tr (E.N.T.); 3 Department of Physiology, Medical Specialty Training Center (TUSMER), Ankara 06420, Turkey

**Keywords:** hematologic malignancies, systemic immune-inflammation index (SII), pan-immune-inflammation value (PIV), prognosis, overall survival, inflammatory markers

## Abstract

*Background and Objectives:* this study aimed to evaluate the prognostic significance of systemic immune-inflammatory markers, particularly the pan-immune-inflammation value (PIV) and systemic immune-inflammation Index (SII), in predicting overall survival among patients with hematologic malignancies. *Materials and Methods:* This retrospective cohort study included 300 patients diagnosed with various hematologic malignancies between January 2020 and January 2025 at the Department of Hematology, Faculty of Medicine, Mersin University. Baseline laboratory data, including neutrophil, lymphocyte, platelet, and monocyte counts, were collected to calculate SII, NLR, PLR, and PIV. Patients were stratified into high and low groups based on the median values of these markers. Overall survival was analyzed using Kaplan–Meier curves and Cox proportional hazards models, adjusted for age, sex, malignancy type, and disease stage. *Results:* High levels of PIV and SII were significantly associated with poorer overall survival. In univariate analysis, high PIV (HR: 2.35, 95% CI: 1.68–3.28, *p* < 0.001) and high SII (HR: 2.12, 95% CI: 1.53–2.95, *p* < 0.001) were strong predictors of mortality. After multivariate adjustment, PIV (adjusted HR: 2.14, 95% CI: 1.47–3.11, *p* < 0.001) and SII (adjusted HR: 1.88, 95% CI: 1.32–2.67, *p* = 0.001) remained independent prognostic factors. Subgroup analyses demonstrated that the predictive power of PIV and SII was consistent across different malignancy types, particularly in acute myeloid leukemia and multiple myeloma patients. *Conclusions:* Our findings indicated that systemic immune-inflammatory markers, particularly PIV and SII, are valuable prognostic tools in hematologic malignancies. These markers, derived from routine blood counts, offer a simple cost-effective means for improving risk stratification. Incorporating these indices into clinical practice could enhance individualized management strategies. Further prospective studies are warranted to validate these findings.

## 1. Introduction

Hematologic malignancies, including leukemias, lymphomas, and multiple myeloma, represent a diverse group of cancers originating from the blood, bone marrow, or lymphatic system. Despite significant advancements in diagnosis and treatment, overall survival (OS) among patients with these malignancies remains variable and is influenced by numerous factors, including disease biology, patient comorbidities, and treatment response [1]. Recent studies have highlighted the pivotal role of systemic inflammation and immune response in cancer progression, suggesting that inflammatory and immune markers may serve as accessible prognostic indicators in hematologic malignancies [2,3,4,5].

Systemic immune and inflammation markers such as the systemic immune-inflammation index (SII), neutrophil-to-lymphocyte ratio (NLR), pan-immune-inflammation value (PIV), and platelet-to-lymphocyte ratio (PLR) have gained attention due to their availability from routine complete blood count (CBC) tests and potential prognostic value [6,7,8,9,10,11,12]. SII (platelet count × neutrophil count/lymphocyte count) reflect the balance between host inflammatory and immune status and has been associated with survival outcomes in various solid tumors [13,14,15,16,17,18,19]. Similarly, elevated NLR and PLR values have been correlated with poor prognosis in both solid and hematologic cancers [20,21,22,23]. More recently, PIV, incorporating lymphocyte, monocyte, platelet, and neutrophil counts, has emerged as a comprehensive marker of systemic inflammation, offering a broader perspective of the host–tumor interaction [21,24].

In hematologic malignancies, systemic inflammation may promote tumor progression by inhibiting antitumor immunity, enhancing angiogenesis, and remodeling the tumor microenvironment [25]. Furthermore, the immune landscape in patients with hematologic cancers is inherently altered due to disease-related immune dysregulation, making immune-inflammatory markers potentially more informative for risk stratification [26,27,28,29].

Despite these insights, the clinical application of these markers in predicting overall survival among hematologic malignancy patients remains insufficiently explored, particularly in real-world settings. Most existing studies have focused on solid tumors, with limited and heterogeneous data regarding hematologic cancers [4,30]. Thus, evaluating the prognostic significance of immune and inflammation markers in this patient population may offer valuable information for risk stratification and personalized treatment planning.

This study aimed to investigate the prognostic value of key immune and inflammation markers, including SII, NLR, PLR, and PIV, in predicting overall survival in patients with hematologic malignancies. By conducting a retrospective analysis, we sought to identify accessible and cost-effective biomarkers that could enhance prognostic assessment in clinical treatments.

## 2. Materials and Methods

### 2.1. Study Design and Study Population

This retrospective cohort study was conducted at the Department of Hematology, Faculty of Medicine, Mersin University. Patient data were collected from hospital records between January 2020 and January 2025. The study protocol was approved by the Mersin University Institutional Review Board, and all procedures conformed to the ethical standards of the Declaration of Helsinki (ethics approval: 78017789/050.01.04/E.1321381, date: 20 February 2020).

### 2.2. Patient Selection, Data Collection, and Outcomes

Patients aged 18 years and older who were diagnosed with hematologic malignancies, including acute myeloid leukemia (AML), acute lymphoblastic leukemia (ALL), chronic lymphocytic leukemia (CLL), non-Hodgkin lymphoma (NHL), Hodgkin lymphoma (HL), and multiple myeloma (MM), were eligible for inclusion. Diagnosis was based on the World Health Organization (WHO) criteria [31].

Exclusion criteria were as follows:

- Presence of active infection at the time of laboratory evaluation.
- History of autoimmune disease or chronic inflammatory disorder.
- Insufficient laboratory or follow-up data.
- Patients who received anti-inflammatory or immunomodulatory therapy within 4 weeks prior to blood sampling.

Based on the recent literature evaluating inflammatory markers in hematologic malignancies, sample sizes ranged between 150 and 400 patients to achieve sufficient statistical power for survival analyses [5,32]. Therefore, aiming for a robust analysis, a target sample size of approximately 300 patients was determined appropriate for this study.

Demographic and clinical data were extracted from the hospital’s electronic medical record system. Collected variables included the following:

- Age, sex.
- Type of hematologic malignancy.
- Stage at diagnosis.
- Treatment regimens.
- Response to therapy (complete remission, partial remission, progression).
- Survival status and follow-up time.

Laboratory parameters were collected at the time of initial diagnosis prior to any chemotherapy or immunotherapy initiation:

- Complete blood count (CBC) parameters: neutrophil count, lymphocyte count, monocyte count, and platelet count.
- Calculation of immune-inflammatory indices:
  ○ Systemic immune-inflammation index (SII): platelet count × neutrophil count/lymphocyte count.
  ○ Neutrophil-to-lymphocyte ratio (NLR): neutrophil count/lymphocyte count.
  ○ Platelet-to-lymphocyte ratio (PLR): platelet count/lymphocyte count.
  ○ Pan-immune-inflammation value (PIV): (neutrophil count × platelet count × monocyte count)/lymphocyte count.

The primary outcome of this study was overall survival (OS), defined as the time from diagnosis to death from any cause or last follow-up.

### 2.3. Statistical Analysis

All statistical evaluations were carried out using SPSS Statistics version 27.0 (IBM Corp., Armonk, NY, USA). The distribution of continuous variables was assessed using the Kolmogorov–Smirnov test. Variables with normal distribution were expressed as mean ± standard deviation (SD), while those not normally distributed were reported as median with interquartile range (IQR). The cohort was stratified into groups based on median values of SII, NLR, PLR, and PIV. Kaplan–Meier survival analysis and log-rank tests were used to compare survival rates between groups. Cox proportional hazards regression models were employed to identify independent predictors of overall survival. Variables with a *p*-value < 0.1 in univariate analyses were included in the multivariate model. A two-tailed *p*-value of <0.05 was considered statistically significant.

## 3. Results

A total of 300 patients with hematologic malignancies were included in this study, with a median age of 58 years and a predominance of male patients (56%). The most common malignancy types were AML, NHL, and MM. Baseline hematological parameters and immune-inflammatory indices, including SII, NLR, PLR, and PIV, were recorded at diagnosis. The median follow-up duration was 24 months. Detailed demographic and laboratory characteristics are summarized in Table 1.

**Figure 1:**
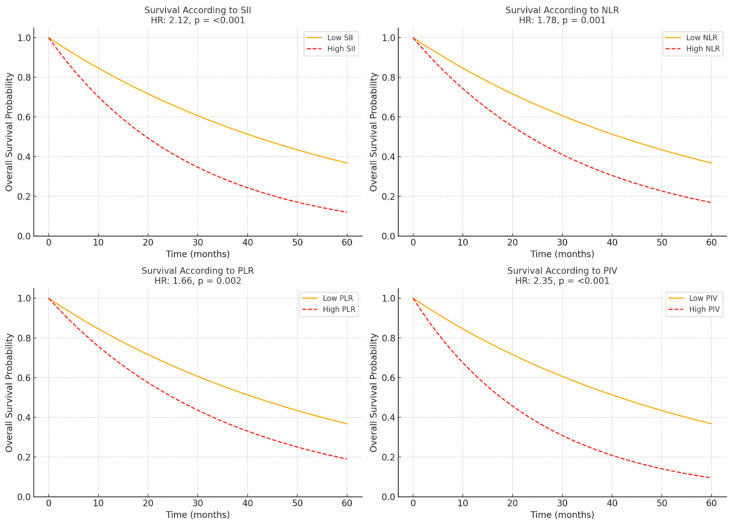
Kaplan–Meier survival curves according to immune-inflammation markers.

Survival analysis based on immune-inflammation markers is shown in Table 2. Patients were divided into high and low groups based on the median values for each marker (750 for SII, 3.25 for NLR, 137.5 for PLR, and 910 for PIV). Patients were categorized into high and low groups according to the median values of SII, NLR, PLR, and PIV. Patients with high SII levels had significantly worse outcomes, with a 2-year overall survival (OS) rate of 43% compared with 65% in the low SII group. The median OS was 20.1 months in the high SII group versus 34.2 months in the low SII group, and high SII was associated with an increased risk of mortality (HR: 2.12, 95% CI: 1.53–2.95, *p* < 0.001). Similarly, high NLR was associated with poorer survival outcomes, with a 2-year OS rate of 46% compared with 68% in the low NLR group (HR: 1.78, 95% CI: 1.29–2.45, *p* = 0.001). Patients with high PLR levels also demonstrated inferior survival (49% vs. 70% 2-year OS), with a hazard ratio of 1.66 (95% CI: 1.20–2.31, *p* = 0.002). Among all markers evaluated, PIV exhibited the strongest prognostic value. The 2-year OS was 41% in the high PIV group compared with 72% in the low PIV group. High PIV was associated with the highest hazard ratio for mortality (HR: 2.35, 95% CI: 1.68–3.28, *p* < 0.001) (Table 2, Figure 1).

Univariate and multivariate Cox regression analyses were performed to evaluate the prognostic significance of immune-inflammatory markers for overall survival, as shown in Table 3. In univariate analysis, high SII (HR: 2.12, 95% CI: 1.53–2.95, *p* < 0.001), high NLR (HR: 1.78, 95% CI: 1.29–2.45, *p* = 0.001), high PLR (HR: 1.66, 95% CI: 1.20–2.31, *p* = 0.002), and high PIV (HR: 2.35, 95% CI: 1.68–3.28, *p* < 0.001) were all significantly associated with worse overall survival. After adjusting for age, sex, malignancy type, and disease stage in the multivariate analysis, high PIV (adjusted HR: 2.14, 95% CI: 1.47–3.11, *p* < 0.001) and high SII (adjusted HR: 1.88, 95% CI: 1.32–2.67, *p* = 0.001) remained as independent predictors of poor survival. In contrast, NLR (*p* = 0.068) and PLR (*p* = 0.087) lost their statistical significance in the multivariate model (Table 3).

Subgroup analyses evaluating the prognostic significance of PIV and SII across different hematologic malignancy types are summarized in Table 4. In patients with AML, both high PIV (HR: 2.52, 95% CI: 1.61–3.95, *p* < 0.001) and high SII (HR: 2.01, 95% CI: 1.32–3.07, *p* = 0.001) were strongly associated with inferior overall survival. Similarly, in patients with NHL, elevated PIV (HR: 2.18, 95% CI: 1.34–3.53, *p* = 0.002) and high SII (HR: 1.76, 95% CI: 1.12–2.78, *p* = 0.015) predicted worse outcomes. Among patients with MM, high PIV showed the strongest association with poor survival (HR: 2.64, 95% CI: 1.52–4.59, *p* < 0.001), and high SII was also a significant predictor (HR: 2.09, 95% CI: 1.21–3.60, *p* = 0.007). These findings confirm that the prognostic value of PIV and SII is consistent across different hematologic malignancy subtypes, with the strongest predictive power observed in AML and MM (Table 4).

## 4. Discussion

In this study, we demonstrated that systemic immune and inflammation markers, specifically PIV and SII, were significant independent predictors of overall survival in patients with hematologic malignancies. Our findings align with the growing body of evidence suggesting that inflammation and immune dysregulation play critical roles in cancer progression and prognosis.

Consistent with our results, Yang et al. showed that a higher SII was associated with worse survival outcomes in patients with solid tumors, proposing SII as a robust marker reflecting the balance between host inflammatory response and immune competence [21]. Similarly, Hu et al. identified SII as a strong prognostic indicator in hepatocellular carcinoma, and its predictive value has since been extended to various malignancies, including hematologic cancers [33]. Our findings reinforce the role of SII, demonstrating its prognostic significance across different subtypes of hematologic malignancies, particularly in AML and MM patients.

PIV, a relatively newer marker combining neutrophil, platelet, monocyte, and lymphocyte counts, has been proposed as a comprehensive indicator of systemic inflammation. Sato et al. first described PIV in colorectal cancer, showing its superior prognostic performance compared with traditional indices like NLR and PLR [34]. Our study is among the few to evaluate PIV in hematologic malignancies, and we observed that a high PIV was strongly and independently associated with poor overall survival. Notably, in our subgroup analysis, PIV retained its prognostic significance across different malignancy types, with the strongest associations observed in AML and MM. This suggests that the immunoinflammatory milieu captured by PIV may play a particularly critical role in the aggressive biological behavior of these diseases.

In contrast, although NLR and PLR were significantly associated with overall survival in univariate analyses, they did not retain independent prognostic significance in the multivariate models. Similar observations were made by Templeton et al., who reported that, while NLR is a useful marker, its prognostic value may be attenuated when adjusted for other clinical and laboratory variables [35]. Qi et al. emphasized that, in hematologic malignancies, simple ratios like NLR and PLR might not fully capture the complexity of the immune landscape, supporting our approach to integrate multiple components into composite indices such as PIV and SII [36].

The biological mechanisms underlying these associations are multifactorial. Neutrophils and platelets are known to promote tumor growth and metastasis through the secretion of pro-inflammatory cytokines and growth factors, while lymphocytes exert antitumor effects through cytotoxic activity. Monocytes contribute to tumor-associated macrophage differentiation, further enhancing tumor progression [26,29,30]. Therefore, an elevated PIV or SII reflects an immune profile characterized by a predominance of pro-tumor inflammation over antitumor immunity, which likely explains the worse outcomes observed.

This study has several strengths. First, it included a relatively large and diverse cohort of patients treated at a single tertiary care center, minimizing inter-institutional variability. Second, it evaluated both traditional and novel immune-inflammatory markers, allowing a comprehensive comparison. Third, it conducted subgroup analyses stratified by malignancy type, which confirmed the consistency of findings across different hematologic cancers.

Given the increasing recognition of the interplay between inflammation, immune response, and cancer progression, future studies should aim to incorporate dynamic monitoring of immune-inflammatory markers throughout the disease course. Additionally, integrating these biomarkers into existing prognostic models or developing new risk stratification systems could improve personalized treatment strategies in hematologic malignancies. Prospective multicenter trials with larger patient populations and longer follow-up periods are warranted to validate the clinical utility of PIV and SII and to explore their potential roles as therapeutic targets in the emerging field of immunomodulation in hematologic cancers.

### Limitations of the Study

However, some limitations must be acknowledged. First, the retrospective design inherently introduces the potential for selection bias and limits the ability to draw definitive causal inferences. Although multivariate analyses were performed to adjust for known confounders such as age, sex, malignancy type, and disease stage, the possibility of residual confounding cannot be entirely excluded. Additionally, this study assessed only baseline immune-inflammatory markers and did not consider their dynamic changes during the course of treatment, which may also carry prognostic significance. Lastly, since the data were derived from a single-center cohort, the generalizability of the findings is limited, and external validation through prospective multicenter studies is warranted to confirm their reproducibility. Moreover, due to the retrospective design, infections that may have occurred during the follow-up period could not be systematically monitored or excluded, which may have affected the prognostic value of the inflammatory marker.

## 5. Conclusions

In conclusion, this study highlighted the significant prognostic value of systemic immune-inflammatory markers, particularly PIV and SII, in patients with hematologic malignancies. Both PIV and SII independently predicted overall survival and demonstrated consistent prognostic utility across different malignancy subtypes, with the strongest associations observed in acute myeloid leukemia and multiple myeloma. These findings suggested that easily accessible and cost-effective markers derived from routine blood tests can provide valuable information for risk stratification and individualized patient management. Further prospective and multicenter studies are necessary to validate these results and to explore the potential integration of immune-inflammatory markers into standard prognostic models for hematologic cancers.

## Figures and Tables

**Table 1:** Demographic and laboratory characteristics of the patients’ variables.

Parameters	Patients (*n* = 300) *n* (%) or Median (Min–Max)
Age (Years)	58 (18–85)
Genders (Male/Female)	168 (56%)/132 (44%)
Type of Malignancy	
Acute Myeloid Leukemia (AML)	102 (34%)
Non-Hodgkin Lymphoma (NHL)	84 (28%)
Multiple Myeloma (MM)	57 (19%)
Acute Lymphoblastic Leukemia (ALL)	30 (10%)
Chronic Lymphocytic Leukemia (CLL)	15 (5%)
Hodgkin Lymphoma (HL)	12 (4%)
Neutrophil count (×109/L)	5.2 (3.8–7.1)
Lymphocyte count (×109/L)	1.6 (1.0–2.3)
Platelet count (×109/L)	220 (150–290)
Monocyte count (×109/L)	0.6 (0.4–0.8)
SII	750 (520–1180)
NLR	3.25 (2.15–5.00)
PLR	137.5 (98–189)
PIV	910 (580–1420)
Follow-up time (Months)	24 (15–38)

NLR—neutrophil-to-lymphocyte ratio; SII—systemic immune-inflammation index; PLR—platelet-to-lymphocyte ratio; PIV—pan-immune-inflammation value.

**Table 2:** Survival analysis based on immune-inflammation markers.

Marker	Group	2-Year Overall Survival (%)	Median OS (Months)	Hazard Ratio (HR) (95% CI)	*p*-Value
SII	Low	65%	34.2	Reference	—
	High	43%	20.1	2.12 (1.53–2.95)	<0.001
NLR	Low	68%	35.7	Reference	—
	High	46%	22.5	1.78 (1.29–2.45)	0.001
PLR	Low	70%	36.0	Reference	—
	High	49%	24.0	1.66 (1.20–2.31)	0.002
PIV	Low	72%	37.8	Reference	—
	High	41%	18.3	2.35 (1.68–3.28)	<0.001

**Table 3:** Univariate and multivariate Cox regression analysis for overall survival.

Variables	Univariate HR (95% CI)	*p*-Value	Multivariate HR (95% CI)	*p*-Value
High SII	2.12 (1.53–2.95)	<0.001	1.88 (1.32–2.67)	0.001
High NLR	1.78 (1.29–2.45)	0.001	1.41 (0.97–2.04)	0.068
High PLR	1.66 (1.20–2.31)	0.002	1.34 (0.96–1.88)	0.087
High PIV	2.35 (1.68–3.28)	<0.001	2.14 (1.47–3.11)	<0.001

Model adjusted for age, sex, malignancy type, and disease stage for multivariate analysis.

**Table 4:** Subgroup analysis: prognostic value of PIV and SII across different hematologic malignancies.

Malignancy Type	Marker	Hazard Ratio (HR) (95% CI)	*p*-Value
AML	High PIV	2.52 (1.61–3.95)	<0.001
AML	High SII	2.01 (1.32–3.07)	0.001
NHL	High PIV	2.18 (1.34–3.53)	0.002
NHL	High SII	1.76 (1.12–2.78)	0.015
MM	High PIV	2.64 (1.52–4.59)	<0.001
MM	High SII	2.09 (1.21–3.60)	0.007

AML—acute myeloid leukemia, NHL—non-Hodgkin lymphoma, MM—multiple myeloma.

## Data Availability

Data are available upon request to the corresponding author.

## References

[1] Siegel R.L., Miller K.D., Wagle N.S., Jemal A. (2023). Cancer statistics, 2023. CA Cancer J. Clin..

[2] Kou J., Huang J., Li J., Wu Z., Ni L. (2023). Systemic immune-inflammation index predicts prognosis and responsiveness to immunotherapy in cancer patients: A systematic review and meta-analysis. Clin. Exp. Med..

[3] Kumar M., Jalota A., Sahu S.K., Haque S. (2024). Therapeutic antibodies for the prevention and treatment of cancer. J. Biomed. Sci..

[4] Ma W., Liu R., Li X., Yu J., Wang W. (2025). Significant association between systemic inflammation response index and prognosis in patients with urological malignancies. Front. Immunol..

[5] Segmen F., Aydemir S., Kucuk O., Dokuyucu R. (2024). The Roles of Vitamin D Levels, Gla-Rich Protein (GRP) and Matrix Gla Protein (MGP), and Inflammatory Markers in Predicting Mortality in Intensive Care Patients: A New Biomarker Link?. Metabolites.

[6] Chu B., Chen Y., Pan J. (2025). Prognostic significance of systemic immune inflammation index for ovarian cancer: An updated systematic review and meta-analysis. J. Ovarian Res..

[7] Zhang Y., Chen Y., Guo C., Li S., Huang C. (2025). Systemic immune-inflammation index as a predictor of survival in non-small cell lung cancer patients undergoing immune checkpoint inhibition: A systematic review and meta-analysis. Crit. Rev. Oncol. Hematol..

[8] Tan Y., Hu B., Li Q., Cao W. (2025). Prognostic value and clinicopathological significance of pre-and post-treatment systemic immune-inflammation index in colorectal cancer patients: A meta-analysis. World J. Surg. Oncol..

[9] Hai-Jing Y., Shan R., Jie-Qiong X. (2023). Prognostic significance of the pretreatment pan-immune-inflammation value in cancer patients: An updated meta-analysis of 30 studies. Front. Nutr..

[10] Yang X.C., Liu H., Liu D.C., Tong C., Liang X.W., Chen R.H. (2022). Prognostic value of pan-immune-inflammation value in colorectal cancer patients: A systematic review and meta-analysis. Front. Oncol..

[11] Peng J., Chen H., Chen Z., Tan J., Wu F., Li X. (2025). Prognostic value of neutrophil-to-lymphocyte ratio in patients with hepatocellular carcinoma receiving curative therapies: A systematic review and meta-analysis. BMC Cancer.

[12] Zhao Y., Wang Y., Jiang Y., Yang J., Zhang Y. (2024). The prognostic impact of neutrophil-to-lymphocyte ratio and platelet-to-lymphocyte ratio on patients with small cell lung cancer receiving first-line platinum-based chemotherapy: A systematic review and meta-analysis. BMC Pulm. Med..

[13] Zhong J.H., Huang D.H., Chen Z.Y. (2017). Prognostic role of systemic immune-inflammation index in solid tumors: A systematic review and meta-analysis. Oncotarget.

[14] Zhang Z., Zhao Y., Wen J., Wang Y., Li J. (2025). Impact of systemic immune-inflammation index and its evaluation of optimal threshold in patients with limited-stage small cell lung cancer: A retrospective study based on 572 cases. Transl. Cancer Res..

[15] Liu F., Yin P., Jiao B., Shi Z., Qiao F., Xu J. (2025). Detecting the preoperative peripheral blood systemic immune-inflammation index (SII) as a tool for early diagnosis and prognosis of gallbladder cancer. BMC Immunol..

[16] Bal O., Acikgoz Y., Yildiz B., Kos F.T., Algin E., Dogan M. (2023). Simple and easily accessible prognostic markers in ewing sarcoma; neutrophil-lymphocyte ratio, neutrophil-platelet score and systemic-inflammation index. J. Cancer Res. Ther..

[17] Yi J., Xue J., Yang L., Xia L., He W. (2023). Predictive value of prognostic nutritional and systemic immune-inflammation indices for patients with microsatellite instability-high metastatic colorectal cancer receiving immunotherapy. Front. Nutr..

[18] Chen X., Hong X., Chen G., Xue J., Huang J., Wang F., Ali W., Li J., Zhang L. (2022). The Pan-Immune-Inflammation Value predicts the survival of patients with anaplastic lymphoma kinase-positive non-small cell lung cancer treated with first-line ALK inhibitor. Transl. Oncol..

[19] Fu F., Deng C., Wen Z., Gao Z., Zhao Y., Han H., Zheng S., Wang S., Li Y., Hu H. (2021). Systemic immune-inflammation index is a stage-dependent prognostic factor in patients with operable non-small cell lung cancer. Transl. Lung Cancer Res..

[20] Howard R., Kanetsky P.A., Egan K.M. (2019). Exploring the prognostic value of the neutrophil-to-lymphocyte ratio in cancer. Sci. Rep..

[21] Yang R., Chang Q., Meng X., Gao N., Wang W. (2018). Prognostic value of Systemic immune-inflammation index in cancer: A meta-analysis. J. Cancer.

[22] Hou Y., Li X., Yang Y., Shi H., Wang S., Gao M. (2023). Serum cytokines and neutrophil-to-lymphocyte ratio as predictive biomarkers of benefit from PD-1 inhibitors in gastric cancer. Front. Immunol..

[23] Guan Y., Xiong H., Feng Y., Liao G., Tong T., Pang J. (2020). Revealing the prognostic landscape of neutrophil-to-lymphocyte ratio and platelet-to-lymphocyte ratio in metastatic castration-resistant prostate cancer patients treated with abiraterone or enzalutamide: A meta-analysis. Prostate Cancer Prostatic Dis..

[24] Bilgin M., Akkaya E., Dokuyucu R. (2024). Inflammatory and Metabolic Predictors of Mortality in Pulmonary Thromboembolism: A Focus on the Triglyceride-Glucose Index and Pan-Immune Inflammation Value. J. Clin. Med..

[25] Ma Q., Hao S., Hong W., Tergaonkar V., Sethi G., Tian Y., Duan C. (2024). Versatile function of NF-kB in inflammation and cancer. Exp. Hematol. Oncol..

[26] Turizo-Smith A.D., Cordoba-Hernandez S., Mejia-Guarnizo L.V., Monroy-Camacho P.S., Rodriguez-Garcia J.A. (2024). Inflammation and cancer: Friend or foe?. Front. Pharmacol..

[27] Fernandes Q., Inchakalody V.P., Bedhiafi T., Mestiri S., Taib N., Uddin S., Merhi M., Dermime S. (2024). Chronic inflammation and cancer; the two sides of a coin. Life Sci..

[28] Nigam M., Mishra A.P., Deb V.K., Dimri D.B., Tiwari V., Bungau S.G., Bungau A.F., Radu A.F. (2023). Evaluation of the association of chronic inflammation and cancer: Insights and implications. Biomed. Pharmacother..

[29] Liu H.M., Cheng M.Y., Xun M.H., Zhao Z.W., Zhang Y., Tang W., Cheng J., Ni J., Wang W. (2023). Possible Mechanisms of Oxidative Stress-Induced Skin Cellular Senescence, Inflammation, and Cancer and the Therapeutic Potential of Plant Polyphenols. Int. J. Mol. Sci..

[30] She S., Shi J., Zhu J., Yang F., Yu J., Dai K. (2024). Impact of inflammation and the immune system on hepatocellular carcinoma recurrence after hepatectomy. Cancer Med..

[31] Attygalle A.D., Karube K., Jeon Y.K., Cheuk W., Bhagat G., Chan J.K.C., Naresh K.N. (2025). The fifth edition of the WHO classification of mature T cell, NK cell and stroma-derived neoplasms. J. Clin. Pathol..

[32] Cristofaro M.G., Ferragina F., Stagliano S., Arrotta A., D’Amico M., Barca I. (2025). Prognostic Value of Systemic Inflammatory Markers in Malignant Tumors of Minor Salivary Glands: A Retrospective Analysis of a Single Center. Cancers.

[33] Hu B., Yang X.R., Xu Y., Sun Y.F., Sun C., Guo W., Zhang X., Wang W.M., Qiu S.J., Zhou J. (2014). Systemic immune-inflammation index predicts prognosis of patients after curative resection for hepatocellular carcinoma. Clin. Cancer Res..

[34] Sato R., Oikawa M., Kakita T., Okada T., Abe T., Tsuchiya H., Akazawa N., Ohira T., Harada Y., Okano H. (2023). A decreased preoperative platelet-to-lymphocyte ratio, systemic immune-inflammation index, and pan-immune-inflammation value are associated with the poorer survival of patients with a stent inserted as a bridge to curative surgery for obstructive colorectal cancer. Surg. Today.

[35] Templeton A.J., McNamara M.G., Seruga B., Vera-Badillo F.E., Aneja P., Ocana A., Leibowitz-Amit R., Sonpavde G., Knox J.J., Tran B. (2014). Prognostic role of neutrophil-to-lymphocyte ratio in solid tumors: A systematic review and meta-analysis. J. Natl. Cancer Inst..

[36] Qi X., Chen J., Wei S., Ni J., Song L., Jin C., Yang L., Zhang X. (2023). Prognostic significance of platelet-to-lymphocyte ratio (PLR) in patients with breast cancer treated with neoadjuvant chemotherapy: A meta-analysis. BMJ Open.

